# Treatment preferences and current practices regarding open tibial shaft fractures

**DOI:** 10.3389/fpubh.2024.1331654

**Published:** 2024-07-05

**Authors:** Shengjun Qian, Yechao Shen, Lingling Sun, Zhan Wang

**Affiliations:** ^1^Department of Orthopedic Surgery, The Second Affiliated Hospital, Zhejiang University School of Medicine, Hangzhou, China; ^2^Orthopedics Research Institute of Zhejiang University, Hangzhou, China; ^3^Key Laboratory of Motor System Disease Research and Precision Therapy of Zhejiang Province, Hangzhou, China; ^4^Zhejiang Provincial Clinical Medical Research Center for Motor System Diseases, Hangzhou, China; ^5^International Chinese Musculoskeletal Research Society, Hangzhou, China

**Keywords:** open tibial shaft fracture, antibiotic prophylaxis, irrigation and debridement, fracture stabilization, wound management

## Abstract

**Purpose:**

The purpose of this study was to reveal the treatment preferences and current practices regarding open tibial shaft fracture (OTSF).

**Patients and methods:**

Online surveys of treatment preferences and current practice of OTSF were conducted by orthopedic trauma doctors from various medical institutions in Zhejiang Province. The survey contains three modules. The first module is the basic information of the participants, the second module is the treatment patterns for Gustilo-Anderson type I-II (GA I/II), and the third module is the treatment patterns for Gustilo-Anderson type IIIA (GA IIIA). Furthermore, each treatment pattern was divided into four aspects, including antibiotic prophylaxis, irrigation and debridement, fracture stabilization, and wound management.

**Results:**

A total of 132 orthopedic trauma doctors from 41 hospitals in Zhejiang province, participated the online surveys. In GA I-IIIA OTSF, more than three-quarters of participants considered <3 h as the appropriate timing of antibiotic administration after trauma. In fact, only 41.67% of participants administered antibiotics within 3 h after trauma. 90.91 and 86.36% of participants thought debridement within 6 h was reasonable for GA I/II and GA IIIA OTSF, respectively. However, in reality only about half of patients received debridement within 6 h on average. The most common reason for delayed debridement was patients’ transport delay. 87.88 and 97.3% of participants preferred secondary internal fixation following external fixation for GA I/II and GA IIIA OTSF, respectively. Additionally, over half of participants preferred use of locking plate for treating GA I-IIIA OTSF. The most common reasons for choosing delayed internal fixation for GA I-IIIA OTSF were infection risk and damage control. 78.79 and 65.91% supported immediate internal fixation after removing the external fixation for GA I-IIIA OTSF, respectively. Regarding wound closure, 86.36 and 63.64% of participants reported primary closure for GA I/II and GA IIIA OTSF, respectively. Over three fourths of participants agreed that preoperative and postoperative multiple wound cultures should be performed to predict infection for GA I-IIIA OTSF.

**Conclusion:**

The study first presents the current preference and practice regarding management of GA I-IIIA OTSF in Zhejiang. Majority of surgeons in our study preferred secondary internal fixation following external fixation for GA I-IIIA OTSF and over half of surgeons preferred use of locking plate for treating GA I-IIIA OTSF. This study may provide a reference for trauma orthopedic surgeons in the treatment of GA I-IIIA OTSF.

## Introduction

The tibia shaft is one of the common sites of open fractures, often resulting from high-energy trauma (1, 2). Compared with open fractures in other sites, the management of open tibial shaft fracture (OTSF) is extremely challenging due to the thin skin soft tissue coverage and poor blood supply along the tibial shaft (3). Despite the rapid development of modern orthopedic treatment techniques, postoperative complications of open tibial fractures are still high (1). The most common complications are infection, nonunion and amputation (4), which seriously affects the quality of life and brings a huge economic burden to individuals and the health system (5). Currently, there are different opinions on the treatment of OTSF. Therefore, how to effectively managing OTSF has always been a difficult problem for orthopedic trauma surgeons.

At present, the most commonly used clinical classification system for open fractures is the Gustilo-Anderson classification system (1984) (6). Initially, this classification system was only used to evaluate open fractures of the tibia, and later it was used to evaluate all open fractures. Because the classification system is simple, effective, and universal, it is widely used by orthopedic trauma doctors. In clinical practices, severe open fractures of tibia are usually considered as Gustilo-Anderson type IIIB and IIIC (GA IIIA/B), while simple open fractures of tibia with mild soft tissue injuries are usually considered as GA I-IIIA (7). External fixation has become a consensus as the preferred fixation method for GA IIIA/B OTSF. However, there is still considerable controversy regarding the treatment patterns for GA I-IIIA OTSF.

To the best of our knowledge, no research on treatment preferences and current practices regarding GA I-IIIA OTSF has been conducted in China.

We took the lead in carrying out relevant surveys of orthopedic trauma surgeons in various medical institutions in Zhejiang Province. We hope this study may provide insight into the management of those fractures.

## Materials and methods

### Survey design and distribution

We conducted a cross-sectional online survey on GA I-IIIA OTSF among orthopedic trauma surgeons in Zhejiang province from October to December 2021. Residents, trainees, and doctors returning from retirement, were excluded. The work has been reported in line with the STROCSS criteria (8). This study was approved by our institutional review board. This study was reviewed and approved by the Ethics Committee of the Second Affiliated Hospital, Zhejiang University School of Medicine (No.2022–0415). These experiments were conducted according to established ethical guidelines, and informed consent obtained from the participants.

Based on solid professional knowledge and extensive literature reading, we made an initial Chinese questionnaire survey. After the initial draft is completed, careful discussion was conducted with experts who are skilled in questionnaire development and related fields of expertise. The questionnaire was modified according to expert opinions, and then a pre-test (ten doctors) was conducted to see if the questions were clear, complete and biased. Finally, we designed the survey by applying mikecrm tool (www.mikecrm.com). The Chinese survey was translated into English version (Supplementary Table S1).

The survey, which was called “Questionnaire survey on treatment preferences and current practices regarding Gustilo-Anderson type I-IIIA open tibial shaft fracture among orthopedic trauma surgeons in Zhejiang Province” comprised three modules. The first module is the basic information of the respondents, including the name and type of medical institution, title, experience in orthopedic trauma, number of OTSF cases treated annually, percentage of hospital admissions within 6 h following injury, and whether the hospital has a set of formal processing procedures for OTSF.

The second module is the treatment patterns for GA I/II, and the third module is the treatment patterns for GA IIIA. Moreover, treatment patterns were divided into four aspects, including (1) antibiotic prophylaxis, (2) irrigation and debridement, (3) fracture stabilization, and (4) wound management. Orthopedic trauma surgeons in active practice were invited to fill in the questionnaire by the email or Wechat App. Participants answered questions based on their clinical experience.

### Statistical analysis

Response data was collected utilizing mikecrm tool and exported to Excel format for further analysis. Percentages for survey results were calculated.

All statistical and descriptive analysis were performed by using SPSS 22.0 software.

## Results

### Demographic information

A total of 132 orthopedic trauma surgeons from 41 hospitals were surveyed in the study. Table 1 shows the demographic information of survey participants. Nearly two thirds of the participants were from third-level hospitals. More than half of participants were associate chief physicians or above. The average years of work in orthopedic trauma for this population were 14 years (1–32 years). Over half of participants treated more than 10 OTSF cases per year. Only 10.61% of participants reported that patients were admitted to hospital within 6 h after trauma. The vast majority of doctors’ respective medical institutions have a set of formal procedures for OTSF.

**Table 1:** Demographic data of the participants.

Variable	*n*	%
**Type of medical institution**		
Second-level B	11	8.33%
Second-level A	40	30.30%
Third-level B	20	15.15%
Third-level A	61	46.21%
**Title**		
Chief physician	23	17.42%
Associate chief physician,	46	34.85%
Attending physician	52	39.39%
Resident physician	11	8.33%
**Experience in orthopedic trauma**		
Mean (years)	14	–
Median (years)	12.5	–
**Number of OTSF cases treated annually**		
0–10	63	47.73%
11–20	38	28.79%
21–30	17	12.88%
31–40	6	4.55%
41–50	3	2.27%
>50	5	3.79%
**Percentage of hospital admissions within 6 h following injury**		
<10%	14	10.61%
10–24%	18	13.64%
25–49%	17	12.88%
50–74%	22	16.67%
75–89%	30	22.73%
>90%	31	23.48%
**A set of formal processing procedures for OTSF**		
Yes	121	91.67%
No	11	8.33%

### Treatment preferences and current practices regarding antibiotic prophylaxis

Table 2 showed the treatment preferences and current practices regarding antibiotic prophylaxis. In terms of route of antibiotic administration, 84.85% preferred intravenous only for GA I/II OTSF, while 28.03% selected intravenous with local antibiotics for GA IIIA OTSF. In both GA I/II and GA IIIA OTSF, more than three-quarters of participants considered <3 h as the appropriate timing of antibiotic administration after trauma. In fact, only 41.67% of participants administered antibiotics within 3 h after trauma. Responses for preferred antibiotic for GA I/II OTSF were 90.15% cephalosporin, 0% aminoglycoside, 1.52% penicillin, 8.33% cephalosporin + aminoglycoside, and 0% other. For GA IIIA OTSF, preferences were 79.55% cephalosporin, 1.52% aminoglycoside, 0.76% penicillin, 17.42% cephalosporin + aminoglycoside, and 0.76% other. For GA I/II OTSF, 39.39% replied the mean duration of antibiotic use after debridement was 2–3 days and 37.88% replied 4–7 days. For GA IIIA OTSF, 40.15% replied the mean duration of antibiotic use after debridement was 4–7 days and 30.3% replied >7 days.

**Table 2:** Treatment preferences and current practices regarding antibiotic prophylaxis.

**Variable**	**Gustilo-Anderson type I-II**	**Gustilo-Anderson type IIIA**
**Route of antibiotic administration**		
Intravenous only	112 (84.85%)	92 (69.7%)
Local antibiotics only	1 (0.76%)	3 (2.27%)
Intravenous and local antibiotics	19 (14.39%)	37 (28.03%)
**Appropriate timing of antibiotic administration after trauma**		
<3 h	105 (79.55%)	104 (78.79%)
3–6 h	23 (17.42%)	24 (18.18%)
6–12 h	3 (2.27%)	3 (2.27%)
>24 h	1 (0.76%)	1 (0.76%)
**Actual average time of antibiotic administration after trauma**		
<3 h	55 (41.67%)	55 (41.67%)
3–6 h	54 (40.91%)	60 (45.45%)
6–24 h	20 (15.15%)	16 (12.12%)
>24 h	3 (2.27%)	1 (0.76%)
**Antibiotic regimen**		
Cephalosporin	119 (90.15%)	105 (79.55%)
Aminoglycoside	0	2 (1.52%)
Penicillin	2 (1.52%)	1 (0.76%)
Cephalosporin + Aminoglycoside	11 (8.33%)	23 (17.42%)
Others	0	1 (0.76%)
**Mean duration of antibiotic use after debridement**		
≤1 day	4 (3.03%)	5 (3.79%)
2–3 days	52 (39.39%)	34 (25.76%)
4–7 days	50 (37.88%)	53 (40.15%)
>7 days	26 (19.70%)	40 (30.3%)

### Treatment preferences and current practices regarding irrigation and debridement

Table 3 showed the treatment preferences and current practices regarding irrigation and debridement. For GA I/II OTSF, the great majority (90.91%) of participants thought debridement within 6 h was reasonable. However, in reality only about half of patients received debridement within 6 h on average. Similar response results for GA IIIA OTSF were observed. Over 60% of participants agreed debridement for GA I-IIIA OTSF could not be appropriately delayed. The most common reason for delayed debridement for GA I/II OTSF was patients’ transport delay (43.8%), followed by inadequate preoperative preparation (33.06%), others (10.33%), surgeon choice/preference (10.33%), and poor self-condition (7.85%). For GA IIIA OTSF, similar causes were found. Regarding average irrigation volume, 36.36 and 35.61% of participants reported using 3 L and 6 L for GA I/II OTSF, respectively. While 31.82% of participants reported using >9 L for GA III OTSF.

**Table 3:** Treatment preferences and current practices regarding irrigation and debridement.

**Variable**	**Gustilo-Anderson type I-II**	**Gustilo-Anderson type IIIA**
**Appropriate timing of operative debridement after trauma**		
<6 h	120 (90.91%)	114 (86.36%)
6–12 h	10 (7.58%)	13 (9.85%)
12–24 h	0	3 (2.27%)
>24 h	0	0
Timing of debridement is unimportant	2 (1.52%)	2 (1.52%)
**Actual average time of operative debridement after trauma**		
<6 h	67 (50.76%)	68 (51.52%)
6–12 h	62 (46.79%)	58 (43.94%)
12–24 h	2 (1.52%)	5 (3.79%)
>24 h	1 (0.76%)	0
Timing of debridement is unimportant	0	1 (0.76%)
**Do you agree debridement for these open fractures can be appropriately delayed?**		
Yes	50 (37.88%)	52 (39.39%)
No	82 (62.12%)	80 (60.61%)
**Reason for delayed debridement (multiple choice)**		
Inadequate preoperative preparation (lack of surgical staff, surgical space, surgical instruments, etc.)	80 (33.06%)	90 (33.58%)
Self-conditions do not allow immediate debridement (multiple injuries, fatal injuries, etc)	19 (7.85%)	33 (12.31%)
Delayed patients transport	106 (43.80%)	109 (40.67%)
Surgeon choice/preference	12 (4.96%)	15 (5.6%)
Others	25 (10.33%)	21 (7.84%)
**Average irrigation volume**		
3 L	48 (36.36%)	37 (28.03%)
6 L	47 (35.61%)	35 (26.52%)
9 L	10 (7.58%)	18 (13.64%)
>9 L	27 (20.45%)	42 (31.82%)

### Treatment preferences and current practices regarding fracture stabilization

Table 4 showed the treatment preferences and current practices regarding fracture stabilization. 87.88% of participants preferred secondary internal fixation following external fixation for GA I/II OTSF and almost all participants (97.3%) prioritized this therapeutic strategy for GA IIIA OTSF. When internal fixation is used, 58.33% of participants preferred to use the locking plate for treating GA I/II OTSF. While 25 and 15.15% of participants selected unreamed and reamed intramedullary nails for GA I/II OTSF, respectively. Similar internal fixation selection pattern was observed in GA IIIA OTSF. If external fixation is used and followed by secondary internal fixation, the great majority (over 90%) of participants selected external fixation bracket for GA I-IIIA OTSF. Reasons for choosing delayed internal fixation for GA I/II OTSF included infection risk (38.66%), damage control (32.59%), poor general condition (24.28%), and others (4.47%). Similar reasons for choosing delayed internal fixation were revealed for GA III OTSF. Furthermore, all participants were asked about the time interval between the installation of the external fixation and the removal of the external fixation. Over two fifths (43.9%) of participants chose both 7–14 days and good inflammatory indicators for GA I/II OTSF. As for GA IIIA OTSF, 27.3 and 18.9% of participants preferred ‘7–14 days with good inflammatory indicators’ and ‘>14 days with good inflammatory indicators’, respectively. When asked if an internal fixation should be installed immediately after the removal of the external fixation, more than three-quarters and 65.91% of the participants agree with this point of view for GA I/II and GA IIIA OTSF, respectively. Additionally, among participants who chose ‘No’ in the previous question, about one third of participants chose 7–14 days and good inflammatory indicators for GA I-IIIA OTSF.

**Table 4:** Treatment preferences and current practices regarding fracture stabilization.

**Variable**	**Gustilo-Anderson type I-II**	**Gustilo-Anderson type IIIA**
**Preferred strategy for fracture fixation**		
Primary internal fixation	13 (9.85%)	2 (1.52%)
Secondary internal fixation following external fixation	116 (87.88%)	125 (94.7%)
External fixation bracket as the final fixation	2 (1.52%)	5 (3.79%)
A cast or brace as the final fixation	1 (0.76%)	0
Others	0	0
**If internal fixation is used, what is the preferred internal fixation method**		
Locking plate	77 (58.33%)	76 (57.58%)
Non-locking plate	2 (1.52%)	4 (3.03%)
Reamed intramedullary nailing	20 (15.15%)	18 (13.64%)
Unreamed intramedullary nailing	33 (25%)	34 (25.76%)
**If secondary internal fixation following external fixation is used, what is the preferred external fixation method**		
External fixation bracket	120 (90.91%)	123 (93.18%)
A cast or brace	5 (3.79%)	5 (3.79%)
Continuous traction	7 (5.3%)	4 (3.03%)
**If secondary internal fixation following external fixation is used, what is the main reason for choosing delayed internal fixation**		
Infection risk	121 (38.66%)	126 (36.52%)
Poor general condition	76 (24.28%)	88 (25.51%)
Damage control	102 (32.59%)	110 (31.88%)
Others	14 (4.47%)	21 (6.09%)
**If secondary internal fixation following external fixation is used, how long is the time interval between the installation of the external fixation and the removal of the external fixation**		
<7 days	24 (10.3%0)	22 (10.73%)
7–14 days	85 (36.48%)	77 (37.56%)
>14 days	37 (15.88%)	39 (19.02%)
Inflammatory indicators are good (erythrocyte sedimentation rate, C-reactive protein, etc)	87 (37.34%)	67 (32.68%)
**If secondary internal fixation following external fixation bracket is used, whether to install the internal fixation immediately after removing the external fixation bracket**		
Yes	104 (78.79%)	87 (65.91%)
No	28 (21.21%)	45 (34.09%)
**The time interval between the removal of the external fixation bracket and the installation of the internal fixation**		
<7 days	5 (12.5%)	12 (18.18%)
7–14 days	13 (32.5%)	22 (33.33%)
>14 days	8 (20%)	11 (16.67%)
Inflammatory indicators are good (erythrocyte sedimentation rate, C-reactive protein, etc.)	14 (35%)	21 (31.82%)

### Treatment preferences and current practices regarding wound management

Regarding wound closure, 86.36 and 63.64% of participants reported primary closure for GA I/II and GA IIIA OTSF, respectively. In the case of primary wound closure, 54.39 and 65.15% of participants chose a VSD negative pressure drainage device for GA I/II and GA IIIA OTSF, respectively. In the case of secondary wound closure, 91.67 and 92.42% of participants chose a VSD negative pressure drainage device for GA I/II and GA IIIA OTSF, respectively. In the case of secondary wound closure, nearly a third of participants believed that the interval between primary debridement and secondary wound closure should be 7–14 days and good inflammatory indicators for GA I-IIIA OTSF. Preoperative and postoperative multiple wound cultures were considered necessary to predict infection for GA I/II OTSF in 78.79 and 82.58% of participants, respectively. For GA IIIA OTSF, the proportion of support for preoperative and postoperative multiple wound cultures increased slightly (Table 5).

**Table 5:** Treatment preferences and current practices regarding wound management.

**Variable**	**Gustilo-Anderson type I-II**	**Gustilo-Anderson type IIIA**
**Primary wound closure**		
Yes	114 (86.36%)	84 (63.64%)
No	18 (13.64%)	48 (36.36%)
**If immediate primary wound closure is used, whether to use VSD negative pressure drainage device for the wound**		
Yes	62 (54.39%)	86 (65.15%)
No	52 (45.61%)	46 (34.85%)
**If delayed wound closure is used, whether to use VSD negative pressure drainage device for the wound**		
Yes	121 (91.67%)	122 (92.42%)
No	11 (8.33%)	10 (7.58%)
**If delayed wound closure is used, how long should it be separated from the debridement?**		
<7 days	48 (24.24%)	41 (20.4%)
7–14 days	73 (36.87%)	79 (39.3%)
>14 days	18 (9.09%)	21 (10.45%)
Inflammatory indicators are good (erythrocyte sedimentation rate, C-reactive protein, etc)	59 (29.8%)	60 (29.85%)
**Multiple wound cultures before debridement to predict infection**		
Yes	104 (78.79%)	108 (81.82%)
No	28 (21.21%)	24 (18.18%)
**Multiple wound cultures after debridement to predict infection**		
Yes	109 (82.58%)	112 (84.85%)
No	23 (17.42%)	20 (15.15%)

## Discussion

OTSF is a major challenge for orthopedic trauma surgeons. The treatment protocol for OTSF varies from country to country, and even from region to region. At present, the treatment procedure of OTSF is still controversial. Additionally, prospective clinical studies on OTSF are lacking. To the best of our knowledge, this is the first comprehensive presentation of treatment preferences and patterns of GA I-IIIA OTSF in China.

In the present study, most medical institutions have established standardized treatment protocols for OTSF, which is in line with high-income countries worldwide (9). However, Albright et al. (9) revealed that only 26.5% of participants reported that their healthcare facilities had standard treatment protocols for OTSF in Latin America. Nearly two-thirds of the orthopedic surgeons surveyed in this study were from high-level teaching hospitals. In addition, over half of participants were experienced orthopedic trauma surgeons and each participant had an average of 10 years of orthopedic trauma clinical experience. 47.7 and 28.79% of participants treated 0–10 and 11–20 OTSF cases per year, respectively. Similar pattern was observed in surveys performed in Latin America (9) and Turkey (10). Our study showed that nearly half of the participants reported that more than 75% of patients were admitted to the hospital within 6 h of injury, while Albright et al. (9) showed that about half of participants were admitted to the hospital within 24 h of injury. Generally, open fractures require emergency surgery, and should be performed within 6 h of injury (11, 12). However, the historic 6-h rule for open fractures was challenged by the early antibiotic administration (12).

### Antibiotic prophylaxis preferences

In view of antibiotic administration method, 28.03% of orthopedic trauma surgeons preferred intravenous with local antibiotics for GA IIIA and 14.39% for GA I/II, which was similar with the results in Latin America (9). Recent literature suggests that local antibiotics may have beneficial effects (13). Timing of antibiotic prophylaxis plays an important role in preventing infection. One previous study found that antibiotic administration over 3 h after injury could lead to an increased risk of infection (14). Moreover, Lack et al. (15) found that antibiotic administration beyond 66 min was an independent predictor of infection for GA III open tibia fractures. Although most of participants preferred <3 h as the appropriate timing of antibiotic administration for GA I-IIIA OTSF. In fact, less than half of the participants were able to administer antibiotics within 3 h after trauma. Thus, how to ensure effective early antibiotic administration is a focus of future research. For GA I/II OTSF, most of participants (90.15%) preferred cephalosporin. For GA IIIA OTSF, 79.55 and 17.42% of participants preferred cephalosporin and cephalosporin + aminoglycoside, respectively. Mundi et al. (16) concluded that a first-generation cephalosporin plus aminoglycoside is suitable for GA III open tibial fractures. For GA I/II OTSF, 39.39 and 37.88% of participants preferred the mean duration of antibiotic use after debridement was 2–3 days and 4–7 days, respectively. For GA IIIA OTSF, 40.15 and 30.30% of participants preferred the mean duration of antibiotic use after debridement was 4–7 days and >7 days, respectively. It’s worth noting that prolonged antibiotic administration (≥3 days) did not achieve the expected benefits for GA I-III open fractures (13), suggesting that prolonged use of antibiotics should be cautious in open fractures.

### Irrigation and debridement preferences

Although debridement within 6 h was not an independent predictor of infection after open fracture (12), most of participants thought debridement within 6 h was reasonable for GA I-IIIA OTSF. In reality, most of them indicated that the actual clinical debridement time was within 12 h after trauma. Albright et al. (9) reported that most of participants performed operative debridement within 24 h in Latin America. A recent meta-analysis revealed that GA III fractures showed an increased risk of infection with progressive delay to debridement (17). However, our study showed that nearly 40% of participants agreed debridement for GA I-IIIA OTSF could be appropriately delayed. Additionally, the most common reasons for delayed debridement were transport delay and inadequate preoperative preparation, which was similar with the findings in Latin America (9). Regarding irrigation volume, 72% of participants reported using 3–6 L for GA I/II OTSF, while 72% of participants reported using >6 L for GA III OTSF. Although there is a lack of clinical trials on irrigation volume, most clinical studies use irrigation strategies of 3, 6, and 9 L normal saline solution bags for the increasing GA types (18).

### Fracture stabilization preferences

Recent clinical studies showed that primary internal fixation is very popular for GA I-IIIA OTSF (7, 19). Albright et al. (9) reported that about half of participants preferred primary internal fixation for GA I/II OTSF and 86% of participants preferred delayed internal fixation for GA III OTSF. Orthopedics and traumatology specialists in Turkey mostly preferred to use intramedullary nail for GA I/II OTSF, and external fixator for GA III OTSF (10). However, some studies have shown that one-stage external fixation following two-stage internal fixation is also a safe and effective treatment strategy for OTSF, which can achieve good biomechanical stability and physical function (20, 21). Moreover, this treatment strategy is beneficial for infection reduction and damage control (21). Interestingly, majority of those surveyed in our study preferred secondary internal fixation following external fixation for GA I-IIIA OTSF. Most clinical studies preferred intramedullary nailing for internal fixation (16). However, our study revealed that over half of participants preferred use of locking plate for treating GA I-IIIA OTSF. The use of a plate may result in better reduction and physical function. Also, if infection occurs, the plate is easier to handle than the intramedullary nail.

When one-stage external fixation following two-stage internal fixation strategy is applied, the great majority (over 90%) of participants preferred external fixation bracket as one-stage external fixation for GA I-IIIA OTSF. Common reasons for delayed internal fixation for GA I-IIIA OTSF were infection risk and damage control. Similarly, over half of participants in Latin America recognized infection risk as the main reason for using delayed internal fixation (9). Furthermore, we found inflammatory biomarkers may be an important reference for the time interval between external fixation installation and removal. For GA I/II open fractures, Ye et al. (21) suggested that internal fixation should be placed as soon as possible when the recovery of general and local conditions is good and the infection is controlled. Our study also showed that immediate internal fixation after removing the external fixation may be indicated for GA I-IIIA OTSF. If immediate internal fixation is not performed, delayed internal fixation can be performed 7–14 days after the removal of the external fixation bracket with good inflammatory indicators.

### Wound management preferences

Previous studies indicated that there is no significant difference in infection rates between primary closure and delayed closure in GA I-IIIA open tibial fractures (22). However, Jenkinson et al. (23) reported that delayed wound closure increased deep-infection rate in GA I-IIIA open fractures. Additionally, Zuelzer et al. (24) found primary wound closure was associated with a decreased infection risk in open tibia fractures. Our study found most of participants preferred primary closure for GA I/II OTSF and nearly two thirds of participants preferred primary closure for GA IIIA OTSF. If the wound closure is delayed, VSD negative pressure drainage device should be used as much as possible for the wound of the primary surgery. Our study suggested that secondary wound closure could be done 7–14 days after primary debridement with good inflammatory indicators. Recently, Islam et al. (25) found multidrug-resistant Gram-negative organisms were increasingly prevalent in orthopedic infection. Combined with the results of this study, we suggest that preoperative and postoperative multiple wound cultures should be performed to predict infection and accurately treat infection for GA I-IIIA OTSF.

There are some limitations of this study. First, the number of orthopaedic surgeons who participated in the questionnaire was relatively small. Second, we only carried out the questionnaire survey in Zhejiang Province. In the future, we will be able to conduct surveys in different provinces at the same time. Third, this questionnaire did not address the treatment preferences and current practices regarding GA IIIB. The strengths of the present study include separate questionnaire for GA I/II and GA IIIB OTSF, and very detailed questions for the whole management process of OTSF.

## Conclusion

This survey first reports Zhejiang orthopaedic trauma surgeons’ treatment preferences and patterns for GA I-IIIA OTSF. Majority of surgeons in our study preferred secondary internal fixation following external fixation for GA I-IIIA OTSF. Further, over half of surgeons preferred use of locking plate for treating GA I-IIIA OTSF. This study can provide evidence and ideas for carrying out relevant clinical research.

## Data availability statement

The raw data supporting the conclusions of this article will be made available by the authors, without undue reservation.

## Ethics statement

The studies involving humans were approved by the Ethics Committee of the Second Affiliated Hospital, Zhejiang University School of Medicine (No.2022–0415). The studies were conducted in accordance with the local legislation and institutional requirements. Written informed consent to participate in this study was not required from the participants in accordance with the national legislation and the institutional requirements.

## Author contributions

SQ: Data curation, Formal analysis, Investigation, Methodology, Project administration, Software, Writing – original draft. YS: Data curation, Investigation, Methodology, Project administration, Writing – review & editing. LS: Investigation, Methodology, Software, Writing – review & editing. ZW: Conceptualization, Funding acquisition, Investigation, Methodology, Project administration, Software, Supervision, Writing – review & editing.
