# Supplementary material for: Treatment preferences and current practices regarding open tibial shaft fractures
Source: Front Public Health. 2024 Jul 5;12:1331654. doi: 10.3389/fpubh.2024.1331654 (PMC11257859; doi:10.3389/fpubh.2024.1331654)
Supplement: Supplementary file 1 [file Data_Sheet_1.docx]

**Questionnaire survey on treatment preferences and current practices regarding Gustilo-Anderson type I-IIIA open tibial shaft fracture among orthopedic trauma surgeons in Zhejiang Province**

**Basic information of the respondents**

| **Variable** |
| --- |
| **Type of medical institution** |
| Second-level B |
| Second-level A |
| Third-level B |
| Third-level A |
| **Title** |
| Chief physician |
| Associate chief physician, |
| Attending physician |
| Resident physician |
| **Experience in orthopedic trauma** |
| Mean （years） |
| Median (years) |
| **Number of OTSF cases treated annually** |
| 0-10 |
| 11 - 20 |
| 21 - 30 |
| 31 - 40 |
| 41 - 50 |
| ＞50 |
| **Percentage of hospital admissions within 6 hours following injury** |
| ＜10% |
| 10%-24% |
| 25%-49% |
| 50%-74% |
| 75%-89% |
| ＞90% |
| **A set of formal processing procedures for OTSF** |
| Yes |
| No |

**Treatment patterns for GA I/II**

| **Variable** | | | | | |
| --- | --- | --- | --- | --- | --- |
| **Route of antibiotic administration** | | | | | |
| Intravenous only | | | | | |
| Local antibiotics only | | | | | |
| Intravenous and local antibiotics | | | | | |
| **Appropriate timing of antibiotic administration after trauma** | | | | | |
| <3 hours | | | | | |
| 3-6 hours | | | | | |
| 6-12 hours | | | | | |
| >24 hours | | | | | |
| **Actual average time of antibiotic administration after trauma** | | | | | |
| <3 hours | | | | | |
| 3-6 hours | | | | | |
| 6-24 hours | | | | | |
| >24 hours | | | | | |
| **Antibiotic regimen** | | | | | |
| Cephalosporin | | | | | |
| Aminoglycoside | | | | | |
| Penicillin | | | | | |
| Cephalosporin + Aminoglycoside | | | | | |
| Others | | | | | |
| **Mean duration of antibiotic use after debridement** | | | | | |
| ≤1 day | | | | | |
| 2-3 days | | | | | |
| 4-7 days | | | | | |
| ＞7 days | | | | | |
| **Appropriate timing of operative debridement after trauma** | | |  |  |  |
| ＜6 hours | | |  |  |  |
| 6-12 hours | | |  |  |  |
| 12-24 hours | | |  |  |  |
| ＞24 hours | | |  |  |  |
| Timing of debridement is unimportant | | |  |  |  |
| **Actual average time of operative debridement after trauma** | | |  |  |  |
| ＜6 hours | | |  |  |  |
| 6-12 hours | | |  |  |  |
| 12-24 hours | | |  |  |  |
| ＞24 hours | | |  |  |  |
| Timing of debridement is unimportant | | |  |  |  |
| **Do you agree debridement for these open fractures can be appropriately delayed?** | | |  |  |  |
| Yes | | |  |  |  |
| No | | |  |  |  |
| **Reason for delayed debridement (multiple choice)** | | |  |  |  |
| Inadequate preoperative preparation (lack of surgical staff, surgical space, surgical instruments, etc.) | | |  |  |  |
| Self-conditions do not allow immediate debridement (multiple injuries, fatal injuries, etc) | | |  |  |  |
| Delayed patients transport | | |  |  |  |
| Surgeon choice/preference | | |  |  |  |
| Others | | |  |  |  |
| **Average irrigation volume** | | |  |  |  |
| 3L | | |  |  |  |
| 6L | | |  |  |  |
| 9L | | |  |  |  |
| ＞9L | | |  |  |  |
| **Preferred strategy for fracture fixation** |  |  |  |  |  |
| Primary internal fixation |  |  |  |  |  |
| Secondary internal fixation following external fixation |  |  |  |  |  |
| External fixation bracket as the final fixation |  |  |  |  |  |
| A cast or brace as the final fixation |  |  |  |  |  |
| Others |  |  |  |  |  |
| **If internal fixation is used, what is the preferred internal fixation method** | | | |  |  |
| Locking plate |  |  |  |  |  |
| Non-locking plate |  |  |  |  |  |
| Reamed intramedullary nailing |  |  |  |  |  |
| Unreamed intramedullary nailing |  |  |  |  |  |
| **If secondary internal fixation following external fixation is used, what is the preferred external fixation method** | | | | |  |
| External fixation bracket |  |  |  |  |  |
| A cast or brace |  |  |  |  |  |
| Continuous traction |  |  |  |  |  |
| **If secondary intenal fixation following external fixation is used, what is the main reason for choosing delayed internal fixation** | | | | |  |
| Infection risk |  |  |  |  |  |
| Poor general condition |  |  |  |  |  |
| Damage control |  |  |  |  |  |
| Others |  |  |  |  |  |
| **If secondary internal fixation following external fixation is used, how long is the time interval between the installation of the external fixation and the removal of the external fixation** | | | | |  |
| ＜7 days |  |  |  |  |  |
| 7-14 days |  |  |  |  |  |
| ＞14 days |  |  |  |  |  |
| Inflammatory indicators are good (erythrocyte sedimentation rate, C-reactive protein, etc) |  |  |  |  |  |
| **If secondary internal fixation following external fixation bracket is used, whether to install the internal fixation immediately after removing the external fixation bracket** | | | | |  |
| Yes |  |  |  |  |  |
| No |  |  |  |  |  |
| **The time interval between the removal of the external fixation bracket and the installation of the internal fixation** | | | | |  |
| ＜7 days |  |  |  |  |  |
| 7-14 days |  |  |  |  |  |
| ＞14 days |  |  |  |  |  |
| Inflammatory indicators are good (erythrocyte sedimentation rate, C-reactive protein, etc) |  |  |  |  |  |
| **Primary wound closure** | |  |  |  |  |
| Yes | |  |  |  |  |
| No | |  |  |  |  |
| **If immediate primary wound closure is used, whether to use VSD negative pressure drainage device for the wound** | |  |  |  |  |
| Yes | |  |  |  |  |
| No | |  |  |  |  |
| **If delayed wound closure is used, whether to use VSD negative pressure drainage device for the wound** | |  |  |  |  |
| Yes | |  |  |  |  |
| No | |  |  |  |  |
| **If delayed wound closure is used, how long should it be separated from the debridement?** | |  |  |  |  |
| ＜7 days | |  |  |  |  |
| 7-14 days | |  |  |  |  |
| ＞14 days | |  |  |  |  |
| Inflammatory indicators are good (erythrocyte sedimentation rate, C-reactive protein, etc) | |  |  |  |  |
| **Multiple wound cultures before debridement to predict infection** | |  |  |  |  |
| Yes | |  |  |  |  |
| No | |  |  |  |  |
| **Multiple wound cultures after debridement to predict infection** | |  |  |  |  |
| Yes | |  |  |  |  |
| No | |  |  |  |  |

**Treatment patterns for GA IIIA**

| **Variable** | | | | | |
| --- | --- | --- | --- | --- | --- |
| **Route of antibiotic administration** | | | | | |
| Intravenous only | | | | | |
| Local antibiotics only | | | | | |
| Intravenous and local antibiotics | | | | | |
| **Appropriate timing of antibiotic administration after trauma** | | | | | |
| <3 hours | | | | | |
| 3-6 hours | | | | | |
| 6-12 hours | | | | | |
| >24 hours | | | | | |
| **Actual average time of antibiotic administration after trauma** | | | | | |
| <3 hours | | | | | |
| 3-6 hours | | | | | |
| 6-24 hours | | | | | |
| >24 hours | | | | | |
| **Antibiotic regimen** | | | | | |
| Cephalosporin | | | | | |
| Aminoglycoside | | | | | |
| Penicillin | | | | | |
| Cephalosporin + Aminoglycoside | | | | | |
| Others | | | | | |
| **Mean duration of antibiotic use after debridement** | | | | | |
| ≤1 day | | | | | |
| 2-3 days | | | | | |
| 4-7 days | | | | | |
| ＞7 days | | | | | |
| **Appropriate timing of operative debridement after trauma** | | |  |  |  |
| ＜6 hours | | |  |  |  |
| 6-12 hours | | |  |  |  |
| 12-24 hours | | |  |  |  |
| ＞24 hours | | |  |  |  |
| Timing of debridement is unimportant | | |  |  |  |
| **Actual average time of operative debridement after trauma** | | |  |  |  |
| ＜6 hours | | |  |  |  |
| 6-12 hours | | |  |  |  |
| 12-24 hours | | |  |  |  |
| ＞24 hours | | |  |  |  |
| Timing of debridement is unimportant | | |  |  |  |
| **Do you agree debridement for these open fractures can be appropriately delayed?** | | |  |  |  |
| Yes | | |  |  |  |
| No | | |  |  |  |
| **Reason for delayed debridement (multiple choice)** | | |  |  |  |
| Inadequate preoperative preparation (lack of surgical staff, surgical space, surgical instruments, etc.) | | |  |  |  |
| Self-conditions do not allow immediate debridement (multiple injuries, fatal injuries, etc) | | |  |  |  |
| Delayed patients transport | | |  |  |  |
| Surgeon choice/preference | | |  |  |  |
| Others | | |  |  |  |
| **Average irrigation volume** | | |  |  |  |
| 3L | | |  |  |  |
| 6L | | |  |  |  |
| 9L | | |  |  |  |
| ＞9L | | |  |  |  |
| **Preferred strategy for fracture fixation** |  |  |  |  |  |
| Primary internal fixation |  |  |  |  |  |
| Secondary internal fixation following external fixation |  |  |  |  |  |
| External fixation bracket as the final fixation |  |  |  |  |  |
| A cast or brace as the final fixation |  |  |  |  |  |
| Others |  |  |  |  |  |
| **If internal fixation is used, what is the preferred internal fixation method** | | | |  |  |
| Locking plate |  |  |  |  |  |
| Non-locking plate |  |  |  |  |  |
| Reamed intramedullary nailing |  |  |  |  |  |
| Unreamed intramedullary nailing |  |  |  |  |  |
| **If secondary internal fixation following external fixation is used, what is the preferred external fixation method** | | | | |  |
| External fixation bracket |  |  |  |  |  |
| A cast or brace |  |  |  |  |  |
| Continuous traction |  |  |  |  |  |
| **If secondary intenal fixation following external fixation is used, what is the main reason for choosing delayed internal fixation** | | | | |  |
| Infection risk |  |  |  |  |  |
| Poor general condition |  |  |  |  |  |
| Damage control |  |  |  |  |  |
| Others |  |  |  |  |  |
| **If secondary internal fixation following external fixation is used, how long is the time interval between the installation of the external fixation and the removal of the external fixation** | | | | |  |
| ＜7 days |  |  |  |  |  |
| 7-14 days |  |  |  |  |  |
| ＞14 days |  |  |  |  |  |
| Inflammatory indicators are good (erythrocyte sedimentation rate, C-reactive protein, etc) |  |  |  |  |  |
| **If secondary internal fixation following external fixation bracket is used, whether to install the internal fixation immediately after removing the external fixation bracket** | | | | |  |
| Yes |  |  |  |  |  |
| No |  |  |  |  |  |
| **The time interval between the removal of the external fixation bracket and the installation of the internal fixation** | | | | |  |
| ＜7 days |  |  |  |  |  |
| 7-14 days |  |  |  |  |  |
| ＞14 days |  |  |  |  |  |
| Inflammatory indicators are good (erythrocyte sedimentation rate, C-reactive protein, etc) |  |  |  |  |  |
| **Primary wound closure** | |  |  |  |  |
| Yes | |  |  |  |  |
| No | |  |  |  |  |
| **If immediate primary wound closure is used, whether to use VSD negative pressure drainage device for the wound** | |  |  |  |  |
| Yes | |  |  |  |  |
| No | |  |  |  |  |
| **If delayed wound closure is used, whether to use VSD negative pressure drainage device for the wound** | |  |  |  |  |
| Yes | |  |  |  |  |
| No | |  |  |  |  |
| **If delayed wound closure is used, how long should it be separated from the debridement?** | |  |  |  |  |
| ＜7 days | |  |  |  |  |
| 7-14 days | |  |  |  |  |
| ＞14 days | |  |  |  |  |
| Inflammatory indicators are good (erythrocyte sedimentation rate, C-reactive protein, etc) | |  |  |  |  |
| **Multiple wound cultures before debridement to predict infection** | |  |  |  |  |
| Yes | |  |  |  |  |
| No | |  |  |  |  |
| **Multiple wound cultures after debridement to predict infection** | |  |  |  |  |
| Yes | |  |  |  |  |
| No | |  |  |  |  |
